# A prospective study of the sensitivity, specificity and diagnostic performance of soluble intercellular adhesion molecule 1, highly sensitive C-reactive protein, soluble E-selectin and serum amyloid A in the diagnosis of neonatal infection

**DOI:** 10.1186/1471-2431-10-22

**Published:** 2010-04-16

**Authors:** J David M Edgar, Vanessa Gabriel, J Ruth Gallimore, Stanley A McMillan, Judith Grant

**Affiliations:** 1 Regional Immunology Service, Royal Hospitals, The Belfast Trust, & School of Medicine, Dentistry & Biomedical Sciences, Queen's University Belfast, Northern Ireland; 2 Department of Neonatal Medicine, Queen's Medical Centre, Nottingham, UK; 3 Department of Medicine, Royal Free & University College Medical School, University College London, London, UK

## Abstract

**Background:**

Diagnosis of neonatal infection is difficult, because of it's non-specific clinical presentation and the lack of reliable diagnostic tests. The purpose of this study was to examine the potential diagnostic value of serum soluble intercellular adhesion molecule-1 (sICAM-1), soluble E-selectin (sE-selectin), highly sensitive C-reactive protein (hsCRP) and serum amyloid A (SAA) measurements, both individually and in combination in the setting of a neonatal intensive care unit.

**Methods:**

219 consecutive serum samples were taken from 149 infants undergoing sepsis work up in a neonatal intensive care unit. Clinical diagnosis was established in a prospective manner, blind to the results of the study measurements. Infants were classified by an experienced paediatrician as infected or not-infected, one week after presentation. Classification was based on clinical presentation, routine laboratory and radiological investigations and response to therapy. The infected group were sub-classified as (a) culture positive infection or (b) culture negative infection. sICAM-1, sE-selectin, hsCRP and SAA levels were determined from stored serum samples after diagnosis was established. Further sub-group analysis of results was undertaken according to early or late onset of infection and preterm or term status. Statistical analysis utilised Mann Whitney U test and ROC curve analysis.

**Results:**

There were significantly increased serum levels of sICAM-1, hsCRP, E selectin (p < 0.001) and SAA (p = 0.004) in infected infants compared with non-infected. ROC curve analysis indicated area under the curve values of 0.79 (sICAM-1), 0.73 (hsCRP), 0.72 (sE-selectin) and 0.61 (SAA). ROC curve analysis also defined optimum diagnostic cut-off levels for each measurement. The performance characteristics of sICAM-1, hsCRP and sE-selectin included a high negative predictive value (NPV) for culture positive infection and this was enhanced by combination of all 4 measurements. Clinical subgroup analysis suggested particularly high NPV for early onset symptoms, however further studies are required to elucidate this finding.

**Conclusions:**

All four study measurements demonstrated some diagnostic value for neonatal infection however sICAM-1, hsCRP and sE-selectin demonstrated the highest NPV individually. The optimum diagnostic cut off level for hsCRP measurement in this study was much lower than currently used in routine clinical practice. Use of a combination of measurements enhanced diagnostic performance, demonstrating sensitivity of 90.3% and NPV of 91.3%. This study suggests there may be value in use of several of these markers, individually and in combination to assist in excluding neonatal infection. Further work is needed to confirm a specific role in the exclusion of early onset infection.

## Background

The diagnosis of infection in preterm infants is difficult, both because of the non-specific clinical presentation and the lack of reliable diagnostic tests [1]. As a result of this uncertainty, antimicrobial chemotherapy is often commenced on the slightest clinical suspicion of infection. This approach, whilst effective in combating acute infection, increases the risks of drug side effects and the emergence of drug resistant organisms within neonatal units. Over recent years there has been great interest in the potential diagnostic value of a range of haematological, and immunological surrogate markers of infection [1,2]. The value of physiological measurements in this context has also recently been examined [3].

C reactive protein (CRP) is a component of the innate immune system and increased levels of CRP are observed early in response to severe bacterial infection. As a classical acute phase reactant, however, CRP elevation alone has insufficient specificity for diagnosis of neonatal infection. Our previous studies indicated that CRP > 6 mg/l and sICAM-1> 300 ng/ml in plasma samples collected in endotoxin free conditions [4,5] were independent predictors of infection with a high sensitivity for clinical infection (95%) and NPV of 97%[4,5]. However such sampling methods are impractical in routine practice and we subsequently demonstrated in a retrospective study, that routinely collected serum samples gave very similar mean levels of sICAM-1[6]. It has also been demonstrated, independent of those studies, that hsCRP measurement below 1 mg/l provides increased sensitivity for neonatal infection [7]. As most diagnostic laboratories do not provide hsCRP measurement, the optimum diagnostic cut off level for CRP/hsCRP remains a matter of debate. E-selectin is a cell adhesion molecule expressed and secreted by activated endothelial cells and is a component of an adhesion cascade that leads to leukocyte and platelet accumulation at sites of inflammation, infection, and/or injury. Levels of soluble E-selectin (sE- selectin) have also been previously documented in neonatal sepsis[8]. Serum Amyloid A (SAA) is also an acute phase protein which has been shown to be of potential diagnostic value in neonatal infection [9].

In this study, we sought to confirm and extend our earlier findings, utilising blood samples drawn in a prospective study, at the time of acute clinical deterioration and prior to antibiotic administration. We chose this time to reflect the moment at which clinicians would require additional laboratory information to guide their decision regarding whether or not, to prescribe antibiotics and were particularly interested to establish whether the high negative predictive values previously obtained were reproduced. Clinical diagnosis was established, by an experienced paediatrician looking after the infants, blinded to the results of the test measurements.

The incorporation of serum amyloid A, sE-selectin and low level hsCRP (< 1 mg/l) in the study, was to establish whether these measurements would enhance diagnostic performance. Specifically, low level hsCRP measurement was included to ascertain whether a new (lower) diagnostic cut off level would be appropriate in this age group rather than the more commonly used level of < 6 mg/l.

We report here the outcome of this study, in particular the performance characteristics of each study measurement regarding the diagnosis of neonatal infection.

## Methods

Premature infants undergoing neonatal intensive care in the Department of Neonatal Medicine, Queen's Medical Centre, Nottingham, England were studied prospectively. This is a regional referral centre based in a University Teaching Hospital which receives infants both from the hospital's obstetric unit and surrounding district general hospitals. At the time of study it received approximately 400 admissions per year.

Infants were recruited consecutively, as they developed acute clinical deterioration. At the time of enrolment into the study, an enrolment form was completed indicating the infant's age, underlying diagnosis, symptoms indicative of acute deterioration, investigations undertaken and therapy (if any) commenced. A peripheral venous blood sample (0.5-1.0 ml) was taken into a plain bottle prior to the administration of antibiotics and transported routinely to the laboratory. Serum was then separated and stored at -30°C until later batch analysis. There were no special storage/handling requirements. Venous blood samples were also taken at the time of routine venepuncture from a number of infants undergoing intensive care with no acute signs of deterioration, as controls.

One week after deterioration, the routinely available investigative results and subsequent clinical course were reviewed (by VG & DE). Investigations commonly used included e.g. full blood count, differential white count, C-reactive protein (>10 mg/L), urine, blood or swab culture, chest or abdominal X-ray and lumbar puncture Infants were classified as infected or non-infected. Clinical outcome beyond one week was not recorded.

Infants were regarded as not infected if (i) their clinical deterioration was transient (ii) a specific non infective cause for the deterioration was identified (iii) there was no haematological, microbiological or radiological evidence of infection and (iv) administration of antibiotics did not result in clinical improvement. The infected group were sub-classified as (a) culture positive infection or (b) culture negative infection, based on the identification of any positive microbiological results from blood, urine, skin, CSF or other cultures. The culture negative infection subgroup included infants in whom there was either haematological or radiological evidence of infection or those who responded significantly to administration of antibiotics. Study measurements (including hsCRP) were not available to the investigators when allocating infants into their groups.

A standardised "panel" of investigations was not used for all infants; rather, investigations were tailored to each individual presentation by the clinician in charge. The investigations undertaken for each infant were recorded. The identification of positive skin swabs, in the absence of any clinical deterioration was insufficient to have an infant classified as infected. Urine samples were obtained by supra-pubic aspiration and lumbar puncture was performed if the clinician felt this was indicated. Interpretation of chest X-ray sought to identify any new areas of pneumonic change. Abdominal X ray was used to identify potential signs of intestinal obstruction or perforation.

Control samples were taken for comparative purposes, however the data from these samples were not utilised in the calculation of diagnostic performance of the test measurements.

sICAM-1 and sE-selectin levels were measured by commercial ELISA (R&D Systems Europe Ltd, Abingdon UK), hsCRP and SAA by highly sensitive automated immunoassays (Abbott IMx).

Statistical analysis utilised non-parametric tests (Mann-Whitney U) from a computerised database (SPSS 11 for windows, ^© ^SPSS inc. 2001). Receiver operating characteristic curves were plotted and analysed to compare diagnostic performance and optimum diagnostic cut off levels (Medcalc^® ^version10.0.2.0 ^© ^Frank Schoonjans).

### Consent

Written informed parental consent was obtained for this study (VG).

### Ethical Approval

Ethical approval for this study was obtained from the Ethics Committee, University Hospital, Queen's Medical Centre, Derby Road, Nottingham, England.

## Results

### Clinical characteristics of study population

219 samples were obtained from 149 infants. 27/219 (13%) of these were control samples and 192/219 were taken from infants who had an acute clinical deterioration.

**Figure 1:**
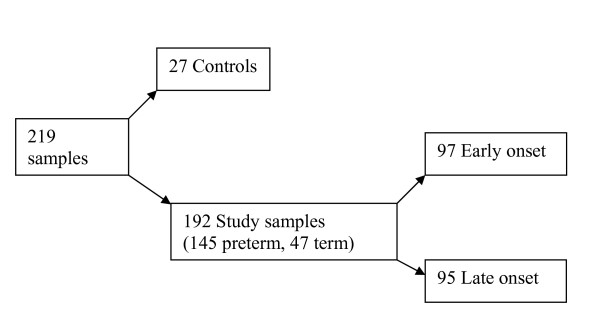
**Initial classification at enrolment to the study**.

145/192 samples were from preterm infants (<36 weeks gestation) and 47/192 from term infants (>36 weeks gestation). 97/192 (50.5%) were suspected of early onset infection (<48 hours after delivery) and 95/192 (49.5%) of late onset infection (> 48 hours after delivery: Figure 1). Gestational age range was from 24-41 weeks. Mean gestational ages and underlying diagnoses that triggered admissions to the neonatal intensive care unit (NICU) are summarised (Table 1). The nature of clinical deterioration prompting investigation for infection and enrolment in the study are summarised in Table 2. The most common presenting clinical feature that triggered sepsis work up was instability of respiration or temperature.

**Table 1:** Gestational age & diagnosis requiring care in NICU

Diagnosis	Infected (n = 74)	Not Infected (n = 114)	Controls (n = 27)
Prematurity/LBW/VLBW/IUGR	33	65	17

Respiratory difficulty (pneumonia, RDS, TTN, BPD, asphyxia, pneumothorax)	23	44	5

NEC/GI obstruction/seizures/jaundice/skin necrosis	7	7	0

Congenital malformation	7	3	3

Suspected infection	17	4	0

Peri-operative care	3	3	0

Haemorrhage/circulatory disturbance (shock, APH, PIH, dehydration, twin-twin transfusion)	0	11	2

PROM, meconium staining, Placenta praevia	11	14	0

**Gestational Age**			

Median	29.0	32.0	32.0

25^th ^quartile	27.0	29.0	28.25

75^th ^quartile	35.0	36.0	34.0

Abbreviations:

RDS: respiratory distress syndrome, NEC: necrotising enterocolitis, PROM: premature rupture of membranes, TTN: transient tachypnoea of the newborn, VLBW: very low birth weight, BPD: bronchopulmonary dysplasia, IUGR: intrauterine growth retardation, PIE: pulmonary interstitial emphysema, APH:ante partum haemorrhage, PIH: primary intracerebral haemorrhage

**Table 2:** Frequency of events prompting investigation for infection

Event	Infected	Not Infected
Unstable respiration	24	77

Unstable temperature	21	17

Rash	12	2

Bradycardia	10	3

Apnoea	6	4

Hypoglycaemia	4	21

Seizure	2	5

Tachycardia	3	1

Acidosis	2	0

Maternal UTI	0	1

Skin Infection	1	0

Smelly baby	0	2

Jaundice	0	2

Hypotension	0	3

Irritability	5	5

NEC	0	1

In terms of "routine" investigations, the following were recorded as undertaken: full blood count (207), CRP (219) blood culture (174), chest x-ray (111), abdominal x-ray (34), urine culture (34), surface swabs (26), lumbar puncture (17), endotracheal tube tip culture (18), gastric aspirate (14), long line culture (8), CT scan (8), amniotic fluid culture (4) and high vaginal swab (4).

**Figure 2:**
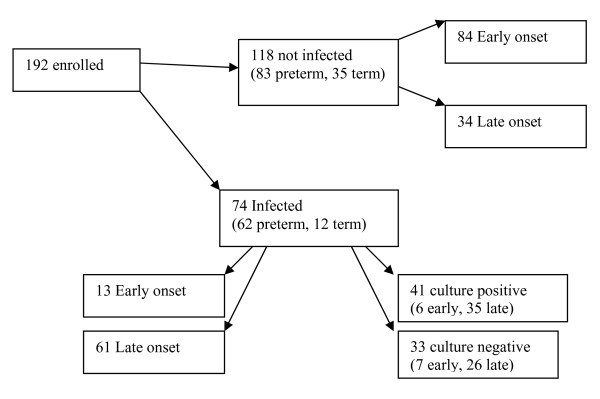
**Classification of samples after one week**.

74/192 (38.5%) samples were clinically classified as infected. 118/192 (61.4%) samples as not infected. 62/74 (83.7%) of infected samples were from preterm infants with 12/74 (16.2%) from term infants. 13/74 (17.6%) were early onset and 61/74(82.4%) were late onset. These included, 41/74 (55.4%) classified as culture positive episodes. 6 of these were early onset (2 pre-term and 4 term) and 35 late onset (31 preterm and 4 term). 33/74 (44.6%) samples were classified as culture negative episodes. 7 of these were early onset (5 preterm and 2 term) and 26 late onset (24 preterm and 2 term) (Figure 2).

### Microorganisms identified

Microorganisms identified in the culture positive infection subgroup (n = 41) included *Staphylococcus epidermidis *(15: 2 early onset, 13 late onset), Coliforms (6: 1 early onset, 5 late onset), Group B Streptococcus (5: 2 early onset, 3 late onset), *Staphylococcus aureus *(5: all late onset), *Enterococcus gallium *(4: all late onset), *Streptococcus viridans *(3: all late onset), other gram positive cocci (2: 1 early onset, 1 late onset), *Haemophilus influenzae *(1: late onset).

In 2/33 culture negative infection samples there was identification of *Enterococcus gallium *(1) and Coliforms (1) from swabs which were regarded as non-pathogenic contamination.

118/192 (61.4%) samples were taken during episodes which, with the benefit of one weeks clinical observation and the results of investigation, were deemed to be non-infectious. 82 of these samples were taken for early onset and 36 for late onset symptoms. In 7/118 cases there was positive cultures regarded as non-pathogenic contamination. (Group B Streptococcus (1), *Staphylococcus epidermidis*(3), *Enterococcus gallium*(1), and Coliforms (2)). For these 118 episodes an alternative explanation, other than infection, was required to explain the acute clinical deterioration and these causes are summarised (Table 3).

**Table 3:** Non infectious causes of clinical deterioration identified in the not-infected infants (n = 118)

Cause	N	Cause	N
RDS	30	Fetomaternal transfusion	2

TTN	18	PROM	2

Unknown	12	IVH	2

Prematurity	7	Hypoglycaemia	2

BPD	7	Hypoxic encephalopathy	2

Secondary apnoea	7	Pneumothorax	1

Asphyxia	5	Steroid leucocytosis	1

GI Obstruction	4	Skin necrosis	1

Dehydration	3	Drug withdrawal	1

IUGR	3	Post operative	1

Meconium staining	2	Duodenal atresia	1

Maternal infection	2	Hypotension	1

		Teratoma	1

			

		Total	118

Abbreviations:

RDS: respiratory distress syndrome, TTN: transient tachypnoea of the newborn, BPD: bronchopulmonary dysplasia, IUGR: intrauterine growth retardation, PROM: premature rupture of membranes IVH: intraventricular haemorrhage.

### Use of antibiotics

Duration of antibiotic therapy was recorded as a surrogate marker of the clinical diagnosis of infection. The maximum duration of antibiotic therapy was 20 days in the culture positive infection subgroup and 10 days in the culture negative infection subgroup. Only one infant in the culture positive infection subgroup was treated for less than 5 days, he died from septicaemia within 24 hours and duration of therapy was therefore recorded for only one day. Five infants in the culture negative infection subgroup were treated for between 2 and 4 days. A single infant in the culture negative infection subgroup was not treated with antibiotics. This was because the diagnosis of culture negative meningitis was only made retrospectively when the infant presented with hydrocephalus some 4 weeks later. 82 infants subsequently classified as not infected were prescribed antibiotics. In 52 these were stopped at 48 hours with 18 receiving 5 days treatment. Summary details of antibiotic therapy duration are provided along with test data (Table 4).

**Table 4:** Study measurements in the clinical groups

Clinical group	*Controls* *(n = 27)*	Not Infected (n = 118)	Infected (n = 74)		
				**Culture positive (n = 41)**	**Culture Negative (n = 33)**

**Duration of antibiotic therapy (days)**	*0* *(0,0)*	2 (1,3)	5 (5,7)	7 (5,7)	5 (5,7)

**sICAM-1 **(ng/ml),	*165 (130,290)*	168 (140,228)	**341** **(236,554)** **P < 0.001**	**405 (252,666)** **P < 0.001**	**306 (230,484)** **P < 0.001**

**hsCRP**(mg/l),	*0.1 (0.1,0.4)*	0.1 (0.1,0.7)	**0.85 (0.3,15.9)** **P < 0.001**	**2.0 (0.7,21.2)** **P < 0.001**	0.45 (0.1,2.2) P = 0.012

**SAA**(mg/l)	*0.9 (0.9,1.0)*	1.0 (0.9,2.0)	1.5 (0.9,5.8) P = 0.004	2.0 (1.0-10.0) P = 0.001	1.0 (0.9,2.0) P > 0.05

**sE-selectin**(ng/ml)	*71 (51,118)*	90 (59,124)	**135** **(94,192)** **P < 0.001**	**158 (94,207)** **P < 0.001**	109 (92,184) P = 0.009

Levels of test measurements in each clinical group and duration of antibiotic therapy is provided. All data quoted as (median, (1^st^, 3^rd ^quartiles)).

Statistical comparison between infected and not infected groups (Mann Whitney U test). Highly significant elevations (P < 0.001)are highlighted in bold. Control samples not included in statistical analysis.

### Results of test measurements in the clinical subgroups

The distribution of the test measurements (sICAM-1, hsCRP, SAA and sE-selectin) was positively skewed and therefore median and 1^st ^& 3^rd ^quartile values are provided for these in each study subgroup (controls(27), not infected(118), infected(74), and culture positive(41) and negative (33) subgroups. Table 4).

The Mann Whitney U test was used to compare the distribution of measurements between the infected and not infected groups. There was no significant difference for any test measurement between the not-infected (n = 118) and the control (n = 27) groups. The infected group was compared to the not infected group, and culture positive and negative subgroups were also compared to the not infected group. Control samples were not utilised in any comparison with samples from infected infants.

There was a highly significant elevation of sICAM-1, hsCRP and sE-selectin (p < 0.001) in both the infected group, and culture positive subgroup, compared with the not infected. SAA was significantly elevated in both groups (infected p = 0.004 and culture positive p = 0.001). For the culture negative subgroup, there was a highly significant elevation of sICAM-1 (p < 0.001), and significant elevation of sE-selectin (p = 0.009) and hsCRP (p = 0.012), but no significant increase in levels of SAA (p > 0.05).

Because some infants suffered repeated deteriorations and were enrolled in the study on several occasions, we further analysed the data to include only one sample/episode per infant. For this analysis, only the first sample from each infant was accepted and the numbers in each group were as follows: control 13, not- infected 89, infected 46 (culture positive 26, culture negative 20) (Table 5). There remained a highly significant increase in sICAM-1, hsCRP and sE-selectin (p < 0.001) in the infected and culture positive subgroup. There was a less significant increase in SAA in the infected (p = 0.021) and culture positive infection subgroup (p = 0.007) compared to and the not-infected. For the culture negative subgroup there was a significant elevation of sICAM-1 (p < 0.001) and hsCRP (p = 0.017) but no significant difference for SAA (p > 0.05) and sE- selectin (p > 0.05).

**Table 5:** Study measurements in the clinical groups excluding repeat samples

Clinical group	*Controls* *(n = 13)*	Not Infected (n = 89)	Infected (n = 46)		
				**Culture positive (n = 26)**	**Culture Negative (n = 20)**
**sICAM-1 **(ng/ml),	*155 (118,228)*	164 (138,200)	**300** **(198,488)** **P < 0.001**	**346** **(215,568)** **P < 0.001**	**268 (190,339)** **P < 0.001**
**hsCRP**(mg/l),	*0.1 (0.1,0.25)*	0.1 (0.1,1.0)	**1.0** **(0.4,15.8)** **P < 0.001**	**1.6 (0.7,20.0)** **P < 0.001**	0.85 (0.1,6.0) P = 0.017
**SAA**(mg/l)	*0.9 (0.9,1.0)*	1.0 (0.9,2.0)	2.0 (0.9,4.8) P = 0.021	2.0 (0.9-5.8) P = 0.007	1.0 (0.9,2.8) P > 0.05
**sE-selectin**(ng/ml)	*61* *(52,101)*	82 (58,120)	**104** **(84,176)** **P < 0.001**	**150** **(93,196)** **P < 0.001**	98 (63,150) P > 0.05

Results excluding repeat samples from individual infants. Only the first episode is included for each infant. Levels of test measurements in each clinical group and duration of antibiotic therapy is provided. All data quoted as (median, (1^st^, 3^rd ^quartiles)). Statistical comparison between infected and not infected groups (Mann Whitney U test). Highly significant elevations (P < 0.001) highlighted in bold. Control samples not included in statistical analysis.

### ROC Curve analysis

**Figure 3:**
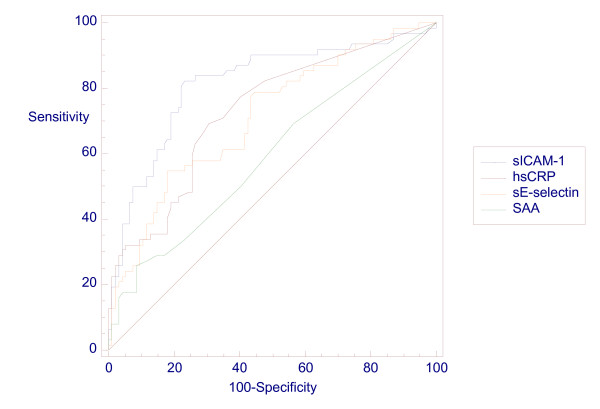
**Receiver operating characteristic (ROC) curve for all test measurements in the diagnosis of infection**.

**Figure 4:**
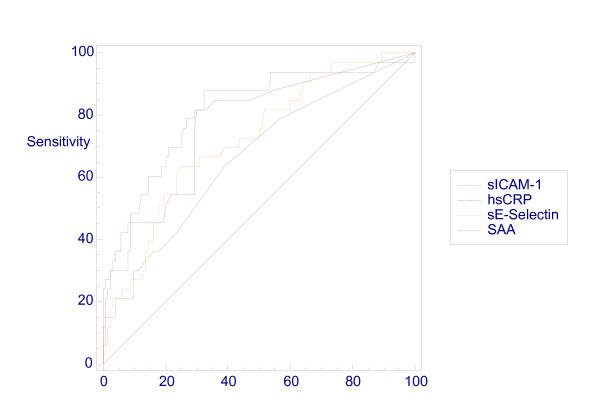
**Receiver operating characteristic (ROC) curve for all test measurements in the diagnosis of culture positive infection**.

Receiver operating characteristics (ROC) curve analysis was utilised to assess diagnostic performance of tests. The area under the curve (AUC) gives an estimate of the percentage of cases correctly classified by the test and values as close as possible to 100% are desirable. The comparative ROC curves for all four measurements for the diagnosis of infection (Figure 3) and for the diagnosis of culture positive infection subgroup (Figure 4) are provided. ROC curve analysis of data utilising only single samples from each infant produced no significant difference in the curves (data not shown). Detailed figures for AUC and other measurements of diagnostic performance (sensitivity, specificity, positive and negative predictive values) are provided in Tables 6 &7. The study populations are also further subdivided and diagnostic performance figures provided for early and late onset infection and preterm and term infants.

**Table 6:** Diagnostic performance of study measurements

Infected					
	**All**	**Early onset**	**Late Onset**	**Preterm**	**Term**

**sICAM-1**	**n = 73**	**n = 12**	**n = 61**	**n = 62**	**n = 11**

**AUC**	**0.79**	**0.56**	**0.66**	**0.80**	**0.65**

Optimum cut off	228 ng/ml	228 ng/ml	368 ng/ml	228 ng/ml	203 ng/ml

sensitivity	76.7%	33.3%	52.5%	82.3%	54.6%

specificity	75.6%	**95%**	72.2%	70.4%	82.4%

Positive predictive value	66.6%	50.3%	76.1%	68.0%	54.6%

Negative predictive value	83.6%	**90.35%**	47.4%	83.8%	82.4%

**hsCRP**	**n = 72**	**n = 13**	**n = 59**	**n = 60**	**n = 12**

**AUC**	**0.73**	**0.74**	**0.64**	**0.73**	**0.78**

Optimum cut off	0.4 mg/l	0.6 mg/l	0.4 mg/l	0.2 mg/l	0.4 mg/l

sensitivity	69.4%	61.5%	71.2%	75%	83.3%

specificity	70.4%	82.3%	55.6%	65.6%	63.6%

Positive predictive value	59.5%	36.3%	71.2%	61.4%	45.4%

Negative predictive value	78.6%	**92.8%**	55.6%	78.2%	**91.3%**

**SAA**	**n = 68**	**n = 12**	**n = 56**	**n = 57**	**n = 11**

**AUC**	**0.61**	**0.66**	**0.59**	**0.59**	**0.69**

Optimum cut off	1 mg/l	1 mg/l	3 mg/l	5 mg/l	1 mg/l

sensitivity	23.5%	58.3%	28.6%	24.6%	81.8%

specificity	**92.8%**	67.1%	**91.4%**	**92.4%**	59.4%

Positive predictive value	66.6%	21.8%	84.2%	70.0%	40.9%

Negative predictive value	66.5%	**91.1%**	44.5%	62.9%	**90.5%**

**sE-selectin**	**n = 62**	**n = 12**	**n = 50**	**n = 52**	**n = 10**

**AUC**	**0.72**	**0.69**	**0.67**	**0.77**	**0.65**

Optimum cut off	132 mg/l	161.7 mg/l	91.5 mg/l	91.5 mg/l	204 mg/l

sensitivity	54.8%	50.0%	71.2%	78.9%	50.0%

specificity	82.3%	**93.9%**	54.8%	67.2%	**93.1%**

Positive predictive value	66.6%	60.2%	71.7%	65.1%	71.3%

Negative predictive value	73.8%	**91.0%**	54.1%	80.4%	84.4%

All infected infants: Area under curve (AUC), optimum diagnostic "cut off" level and diagnostic performance statistics derived from ROC curve analysis for each test measurement for all infected infants and subcategories defined as early/late onset symptoms, and preterm/term infants.

**Table 7:** Diagnostic performance of study measurements for culture positive infection

Culture positive infection					
	**All**	**Early onset**	**Late Onset**	**Preterm**	**Term**

**sICAM-1**	**n = 40**	**n = 5**	**n = 35**	**n = 33**	**n = 7**

**AUC**	**0.78**	**0.57**	**0.65**	**0.81**	**0.56**

Optimum cut off	249 ng/ml	228 ng/ml	504 ng/ml	249 ng/ml	170 ng/ml

sensitivity	77.5%	40.0%	48.6%	87.9%	71.4%

specificity	66.9%	**93.0%**	79.0%	60.0%	55.3%

Positive predictive value	38.7%	25.0%	56.7%	39.7%	22.7%

Negative predictive value	**91.7%**	**96.4%**	73.1%	**94.3%**	**91.3%**

**hsCRP**	**n = 40**	**n = 6**	**n = 34**	**n = 32**	**n = 8**

**AUC**	**0.78**	**0.81**	**0.71**	**0.79**	**0.72**

Optimum cut off	0.6 mg/l	0.6 mg/l	0.6 mg/l	0.6 mg/l	0.4 mg/l

sensitivity	80%	83.3%	79.4%	81.2%	87.5%

specificity	71.4%	80.2%	59.0%	73.6%	59.5%

Positive predictive value	43.2%	22.7%	51.9%	47.3%	31.8%

Negative predictive value	**92.9%**	**98.6%**	83.7%	**93.1%**	**95.7%**

**SAA**	**n = 36**	**n = 5**	**n = 31**	**n = 29**	**n = 7**

**AUC**	**0.68**	**0.74**	**0.67**	**0.68**	**0.65**

Optimum cut off	1 mg/l	1 mg/l	0.9 mg/l	3 mg/l	1 mg/l

sensitivity	63.9%	80.0%	77.4%	41.4%	85.7%

specificity	64.3%	47.0%	46.7%	86.9%	55.6%

Positive predictive value	31.1%	12.5%	42.9%	46.2%	27.3%

Negative predictive value	87.6%	**98.2%**	80.0%	84.5%	**95.2%**

**sE-selectin**	**n = 33**	**n = 5**	**n = 28**	**n = 27**	**n = 6**

**AUC**	**0.72**	**0.77**	**0.66**	**0.79**	**0.52**

Optimum cut off	132 mg/l	162 mg/l	116 mg/l	132 mg/l	224 mg/l

sensitivity	63.6%	60.0%	67.9%	66.7%	33.3%

specificity	76.0%	**90.3%**	62.3%	79.4%	87.9%

Positive predictive value	41.2%	30.0%	48.7%	48.6%	40.0%

Negative predictive value	88.8%	**97.0%**	78.6%	89.0%	88.2%

Culture positive infected infants: Area under curve (AUC), optimum diagnostic "cut off" level and diagnostic performance statistics derived from ROC curve analysis for each test measurement for culture positive infected infants and subcategories defined as early/late onset symptoms, and preterm/term infants.

The ranking of the tests for diagnosis of all infection by area under the curve was sICAM-1 = 0.79, hsCRP = 0.73, sE-selectin = 0.72, SAA = 0.61 (Table 6). Ranking order of tests was identical and AUC almost identical (sICAM-1 = 0.78, hsCRP = 0.78, sE-selectin = 0.72, SAA = 0.68) for the diagnosis of culture positive infection (Table 7). Optimum diagnostic "cut-off" levels were identified from the ROC curves for sICAM-1 (> 228 ng/ml), hsCRP (> 0.4 mg/l) sE-selectin (>132 ng/ml) and SAA (>1 mg/l) (Table 6). Diagnostic performance figures are also provided for a combination of all test measurements (Table 8) and for a combination of sICAM-1 and hsCRP (Table 9)

**Table 8:** ROC curve analysis statistics for combination of all 4 test measurements

Infected					
	**All**	**Early onset**	**Late onset**	**Preterm**	**Term**

**sICAM-1+hsCRP** **+SAA+sE selectin**	**n = 62**	**n = 12**	**n = 50**	**n = 52**	**n = 10**

**AUC**	**0.84**	**0.72**	**0.74**	**0.84**	**0.78**

sensitivity	**90.3%**	66.7%	66.0%	**92.3%**	60.0%

specificity	67.0%	87.3%	74.2%	69.2%	89.7%

Positive predictive value	64.4%	50.0%	80.5%	70.6%	66.7%

Negative predictive value	**91.3%**	**93.2%**	57.5%	**91.8%**	86.7%

**Culture positive infection**					

	**All**	**Early onset**	**Late Onset**	**Preterm**	**Term**

**sICAM-1+hsCRP** **+SAA+sE selectin**	**n = 33**	**n = 5**	**n = 28**	**n = 27**	**n = 6**

**AUC**	**0.84**	**0.82**	**0.75**	**0.87**	**0.63**

sensitivity	84.8%	80.0%	50.0%	**96.3%**	33.3%

specificity	66.7%	82.9%	88.7%	65.6%	**97.0%**

Positive predictive value	40.6%	25.0%	70.0%	45.6%	66.7%

Negative predictive value	**94.3%**	**98.3%**	77.0%	**98.3%**	88.9%

**Table 9:** ROC curve analysis statistics for combination of sICAM-1 + hsCRP measurements

Infected					
	**All**	**Early onset**	**Late Onset**	**Preterm**	**Term**

**sICAM-1 + hsCRP**	**n = 71**	**n = 12**	**n = 59**	**n = 60**	**n = 11**

**AUC**	**0.81**	**0.56**	**0.68**	**0.82**	**0.65**

sensitivity	78.9%	33.3%	59.3%	85.0%	45.4%

specificity	75.9%	**96.0%**	69.4%	70.0%	**90.6%**

Positive predictive value	67.5%	57.1%	76.1%	68.0%	62.5%

Negative predictive value	85.0%	**90.1%**	51.0%	86.2%	82.9%

**Culture positive infection**					

	**All**	**Early onset**	**Late Onset**	**Preterm**	**Term**

**sICAM-1 + hsCRP**	**n = 39**	**n = 5**	**n = 34**	**n = 32**	**n = 7**

**AUC**	**0.79**	**0.583**	**0.68**	**0.82**	**0.57**

sensitivity	71.8%	40.0%	52.9%	85.0%	71.4%

specificity	75.0%	**95.2%**	76.7%	70.0%	58.3%

Positive predictive value	43.7%	33.3%	58.1%	68.0%	25.0%

Negative predictive value	**90.8%**	**96.3%**	75.0%	86.2%	**91.3%**

## Discussion and Conclusions

This prospective study was designed to confirm the hypothesis that measurement of sICAM-1 in routinely collected serum samples was of diagnostic value in neonatal infection. We also wished to test the additional value of low level hsCRP, serum amyloid A (SAA) and sE-selectin measurements as studies by other groups had suggested these may have clinical utility.

Since this study was commenced, Ng has made recommendations for optimum study design [10] the majority of which we fulfilled. This study was prospective, infants were recruited consecutively, and criteria for inclusion in the study are documented. The definition of infection was "infected" versus "non-infected" with the former subdivided into "culture positive" and "culture negative" infection subgroups. Data analysis included use of ROC curves and calculation of predictive values. The two areas where we departed from Ng's guidelines were (i) including early and late onset infection and preterm and term infants and (ii) allowing more than one episode of clinical infection per infant.

With regard to the first point, analysis of our data indicates that we recruited almost exactly 50% early and 50% late onset infection (Figure 1). However, confirmation of early onset infection was much less common (13/97) than late onset disease (61/95) (Figure 2). We have provided separate diagnostic performance figures for these subgroups in Tables 6 &7, however because the numbers of term infants and early onset infection in the infected group are both small, the diagnostic performance data for these subgroups should be interpreted cautiously. In relation to point (ii) we did include more than one suspected infected episode per infant, however data analysis did not indicate any significant effect on the results obtained as a result (Tables 4 &5).

Finally, Ng also recommended the future use of multiplexed analysis of multiple measurements for the diagnosis of neonatal infection. The data presented here supports the use of a combination of test to improve diagnostic performance and we have recently published studies supporting that view [11].

With regard to recruitment in this study, we believe that the range of underlying diagnoses requiring admission to intensive care, the symptoms triggering investigation and organisms identified are consistent with routine clinical practice. 27 control samples were obtained from infants undergoing intensive care who were clinically stable. It was reassuring that no significant difference was found in the levels of any of the study measurements between these samples and the 118 samples obtained from infants identified as not infected. This suggests that these measurements are not subject to non-specific modulation by routine intensive care processes. It is important to note however that these control samples were not utilised in any further statistical analysis.

Classification of infants as infected or not-infected is at times difficult and we have endeavoured to be as clear as possible in how we approached this process which is crucial to evaluating our study. We believe that strength of this study is that the classification of infants as infected or not infected was made by an experienced paediatrician directly involved in the care of the infants in the light of one weeks follow up after the initial presentation with acute clinical deterioration. Classification was made in the knowledge of the routinely available investigations but blinded to the study results. As highlighted in the results section, the identification of a positive culture from a biological sample was insufficient of itself to result in the classification of an infant as infected. We have provided the non-infectious causes of acute clinical deterioration, where this was identifiable and also included duration of antibiotic therapy as a surrogate marker of infection status. A number of clinical factors and routine investigations were taken into account in classification, however we must recognise that this is to some extent an inexact science and despite our best efforts, it is always possible for a small number of infants to have been misclassified.

All four study measurements were significantly elevated in the infected samples (n = 74) compared with the non-infected (n = 118) and controls (n = 27). This suggested that all four may have some diagnostic utility. In considering diagnostic performance, we preferentially utilised AUC as an indicator of the percentage of infants correctly classified and from this confirmed the optimum diagnostic cut off level as the point on the curve closest to the upper left corner, providing maximum sensitivity and specificity. However, laboratory tests may be used to identify or to exclude a diagnosis and the mode of use would affect the selection of a diagnostic cut off level. It is important to remember that as different cut off points are used and sensitivity of an assay increases, in most cases specificity falls, leading to increased "false positive" results and vice-versa.

SAA measurement was the least significantly elevated in the infected samples (p = 0.004) overall and ROC curve analysis confirmed SAA as the least discriminatory of the four study measurements (AUC 0.61). sE-selectin measurement was highly significantly elevated in both the infected samples overall and the culture positive subgroup (both p < 0.001) as was hsCRP measurement. ROC curve analysis indicated that hsCRP measurement was slightly more discriminating than sE-selectin for both overall infection (AUC 0.73 vs 0.72) and the culture positive infection subgroup (AUC 0.78 vs 0.72).

The optimum diagnostic cut-off for hsCRP was 0.4 mg/l for infection and 0.6 mg/l for the culture positive infection subgroup. This confirms earlier work indicating that low level hsCRP measurement is of diagnostic value and therefore diagnostic laboratories should consider providing this more sensitive assay in this clinical situation [7]. Finally, sICAM-1 measurement was highly significantly elevated in both the infected samples overall and both the culture positive and negative subgroups (all p < 0.001). ROC curve analysis indicated that sICAM-1 measurement correctly classified most infants in this study (AUC 0.79 for all infected samples and 0.78 for the culture positive subgroup).

ROC curve analysis of combinations of tests suggests that this results in improved diagnostic performance for neonatal infection.(Tables 8 &9). The combination of all 4 study measurements (Table 8) indicated highest AUC of the study at 0.84 for both infection and the culture positive subgroup. The combination of sICAM-1 and hsCRP measurement resulting in AUC of 0.81 and 0.79 for all infection and the culture positive subgroup respectively.

An important finding in our previous work was that of a high negative predictive value for infection for a combination of sICAM-1 and CRP. In this study, sICAM-1, CRP and sE-selectin gave negative predictive value (NPV) for infection of 83.6%, 78.6% and 73.8% respectively (Table 6) These figures rose 91.7%, 92.9% and 88.8% respectively for culture positive infection. The combination of all 4 measurements provides a NPV of 91.3% for infection and 94.3% for culture positive infection in this study (Table 8). These figures exceed the performance of a combination of sICAM-1 and CRP alone (Table 9). The analysis of diagnostic performance of the early/late onset and preterm/term babies is of interest, despite the small numbers involved. It appears there may be particular value in these measurements in the exclusion of early onset infection, however further studies specifically designed to test this hypothesis would be required.

In this study we have therefore confirmed prospectively that all four test measurements are significantly elevated in the serum of infected infants. Results were obtained from routinely collected samples. The optimum diagnostic levels for these tests have been established and data indicate that for CRP, this level is well below that which is routinely available in diagnostic laboratories, suggesting that the introduction of hsCRP assays should be considered. The use of combinations of several measurements clearly has benefit in improving diagnostic performance and in particularly assisting in the exclusion of infection as a cause of acute clinical deterioration.

The ability to exclude infection in infants undergoing intensive care is clinically extremely important. NPV levels achieved in this study would be very helpful to the clinician in making the decision as to whether it was safe to withhold or discontinue antibiotic therapy. Further studies should focus on the use of combinations of likely candidate markers of infection to develop combinations suitable for use in the acute neonatal intensive care setting.

## Competing interests

The authors declare that they have no competing interests.

## Authors' contributions

DE, VG and JG conceived and designed the study. VG undertook enrolment and consent. VG and JG were responsible for clinical management of the infants including establishment of diagnosis. DE and VG undertook weekly supervision and data collection. RG and SAMcM undertook laboratory analysis of study parameters. DE drafted the paper which all authors read and approved.

## Pre-publication history

The pre-publication history for this paper can be accessed here:

http://www.biomedcentral.com/1471-2431/10/22/prepub
